# Unraveling the benefits of *Bacillus subtilis* DSM 29784 poultry probiotic through its secreted metabolites: an *in vitro* approach

**DOI:** 10.1128/spectrum.00177-24

**Published:** 2024-09-17

**Authors:** Nuria Vieco-Saiz, Damien P. Prévéraud, Eric Pinloche, Aurélien Morat, Pauline Govindin, Hervé M. Blottière, Elliot Matthieu, Estelle Devillard, Jessika Consuegra

**Affiliations:** 1 European Laboratory of Innovation Science & Expertise (ELISE). Adisseo France S.A.S., Saint Fons, France; 2 Adisseo France S.A.S., Antony, France; 3 MGP Metagenopolis, INRAE, Université Paris-Saclay, Jouy-en-Josas, France; 4 Nantes Université, INRAE, UMR 1280, PhAN, Nantes, France; University of California Davis, San Bernardino, California, USA

**Keywords:** *Bacillus subtilis *29784, niacin, hypoxanthine, pantothenate, probiotic metabolites, microbiota, intestinal health, inflammation, animal resilience

## Abstract

The probiotic *Bacillus subtilis* 29784 (Bs29784) sustains chicken’s intestinal health, enhancing animal resilience and performance through the production of the bioactive metabolites hypoxanthine (HPX), niacin (NIA), and pantothenate (PTH). Here, using enterocyte *in vitro* models, we determine the functional link between these metabolites and the three pillars of intestinal resilience: immune response, intestinal barrier, and microbiota. We evaluated *in vitro* the capacity of Bs29784 vegetative cells, spores, and metabolites to modulate global immune regulators (using HT-29-NF-κB and HT-29-AP-1 reporter cells), intestinal integrity (HT-29-MUC2 reporter cells and Caco-2 cells), and cytokine production (Caco-2 cells). Finally, we simulated intestinal fermentations using chicken’s intestinal contents as inocula to determine the effect of Bs29784 metabolites on the microbiota and their fermentation profile. Bs29784 vegetative cells reduced the inflammatory response more effectively than spores, indicating that their benefit is linked to metabolic activity. To assess this hypothesis, we studied Bs29784 metabolites individually. The results showed that each metabolite had different beneficial effects. PTH and NIA reduced the activation of the pro-inflammatory pathways AP-1 and NF-κB. HPX upregulated mucin production by enhancing MUC2 expression. HPX, NIA, and PTH increased cell proliferation. PTH and HPX increased epithelial resilience to an inflammatory challenge by limiting permeability increase. In cecal fermentations, NIA increased acetate, HPX increased butyrate, whereas PTH increased acetate, butyrate, and propionate. In ileal fermentations, PTH increased butyrate. All molecules modulated microbiota, explaining the different fermentation patterns. Altogether, we show that Bs29784 influences intestinal health by acting on the three lines of resilience via its secreted metabolites.

**IMPORTANCE:**

Probiotics provide beneficial metabolites to its host. Here, we describe the mode of action of a commonly used probiotic in poultry, Bs29784. By using *in vitro* cellular techniques and simulated chickens’ intestinal model, we show the functional link between Bs29784 metabolites and the three lines of animal resilience. Indeed, both Bs29784 vegetative cells and its metabolites stimulate cellular anti-inflammatory responses, strengthen intestinal barrier, and positively modulate microbiota composition and fermentative profile. Taken together, these results strengthen our understanding of the effect of Bs29784 on its host and explain, at least partly, its positive effects on animal health, resilience, and performance.

## INTRODUCTION

With the global trend of eliminating or restricting antibiotic growth promoters in animal feed (1), probiotics have emerged as a significant alternative to enhance animal performance (2). *Bacillus* species, in particular, are advantageous due to their resistance to heat, chemical stresses, and acidic conditions in the gastric fluids (3, 4), making them suitable for the livestock industry where feed undergoes extreme processing conditions (5). *Bacillus* spores remain dormant in the feed and activate upon ingestion in the gastrointestinal tract (GIT) of chickens (6), where they produce beneficial metabolites such as vitamins, antimicrobial peptides, exopolysaccharides, and enzymes (7, 8). Specifically, *Bacillus subtilis* DSM 29784 (Bs29784) has been developed to improve intestinal health and performance in broilers, particularly under necrotic enteritis conditions (9–11). Bs29784 has been shown to enhance intestinal health by increasing microvilli length and the relative abundance of butyrate-producing genera *Ruminococcus* and *Lachnoclostridium*, resulting in improved broiler performance (10).

Further research revealed that Bs29784 modulates IL-8 production in response to several *in vitro* pro-inflammatory stimuli (12). These anti-inflammatory properties are probably related to the reduction of NF-κB-dependent iNOS upregulation upon exposure to pro-inflammatory cytokines, likely due to the sequestration of NF-κB in the cytosol (12). A series of experiments aiming to investigate the metabolites of Bs29784 have been reported. Kruse and colleagues (13) detected a high level of antioxidants and bioactive substances such as surfactin in the supernatant of Bs29784 culture using high-performance thin-layer chromatography (HPTLC). Choi et al. (14) identified an increase in specific metabolites produced by Bs29784 in the gut of 13-day-old broiler chickens *in vivo*. Pantothenate (PTH), niacin (NIA), and hypoxanthine (HPX) were key metabolites consistently secreted by the probiotic in their metabolomic trial. Concentrations of these metabolites correlated with increased *Bacillus* spp. in various intestinal sections. Moreover, the families *Ruminococcaceae* and *Lachnospiraceae* were significantly associated with nicotinic acid levels in the cecum. These molecules are well known for their benefits on the host. PTH, also known as vitamin B5, is an essential precursor of coenzyme A (15). It acts as a cofactor or direct precursor for numerous cellular intermediates of neurotransmitter synthesis and fatty acid oxidation. NIA, or vitamin B3, is essential for a large number of metabolic pathways as redox cofactor (15). HPX is a purine that promotes epithelial cellular function in the gut (16). Hence, we hypothesized that these metabolites contribute to Bs29784 benefits to the host and its microbiome by influencing the three lines of intestinal resilience: immune response, gut barrier, and intestinal microbiome. To test this hypothesis, we conducted *in vitro* evaluations of the barrier integrity and anti-inflammatory properties of Bs29784 forms and their metabolites. Using established cellular models as HT-29 reporter cell lines (17), we investigated the activation and repression of relevant global transcription factors known to modulate inflammatory responses, NF-κB and AP-1. Furthermore, we measured enterocyte cytokine release upon an inflammatory challenge and maintenance of the intestinal barrier (transepithelial electrical resistance [TEER] measurements) using Caco-2 cells, a widely used *in vitro* cellular model for studying intestinal epithelial barrier (18, 19). Moreover, we assessed the impact of Bs29784 metabolites on intestinal microbial ecology through the simulation of ileal and cecal fermentations using of 28-day-old broiler intestinal contents as starter inocula.

Altogether, our study suggests that metabolites produced by Bs29784, including PTH, NIA, and HPX, play a significant role, at least *in vitro*, in modulating inflammatory pathways, mucin production, and microbiota composition. These actions may contribute to the improved *in vivo* intestinal health reported on broilers supplemented with Bs29784 (10).

## RESULTS

Probiotic spores are a dormant form of bacteria exhibiting minimal metabolism. When they germinate, they enter into a metabolically active vegetative state, which is frequently correlated with probiotic’s biological functions. Here, we aimed to characterize the effect of the different forms of the probiotic Bs29784 and its metabolites on intestinal homeostasis. First, we assessed their effects on global transcription factors, using reporter cell lines, and their downstream consequences on cell viability, gut integrity, and interleukin release. Furthermore, we analyzed the related microbiota modifications triggered by Bs29784’s metabolites.

### Bs29784 vegetative cells have a preventive effect against an inflammatory challenge

To assess the preventive and the curative effect of Bs29784 and its metabolites on immune regulation, we measured their capability to modulate the activation of the global transcription factor NF-κB using HT-29 cells stable transfected with a plasmid containing the secreted alkaline phosphatase (SEAP) gene under the control of NF-κB binding elements. In the preventive model, HT-29 cells were pre-incubated for 1 hour with probiotic Bs29784 forms or its metabolites before the pro-inflammatory challenge induced by TNF-α. In the curative model, the cells were exposed to the pro-inflammatory challenge 1 h before the treatment (probiotic Bs29784 forms or its metabolites) was added into the well.

**Fig 1:**
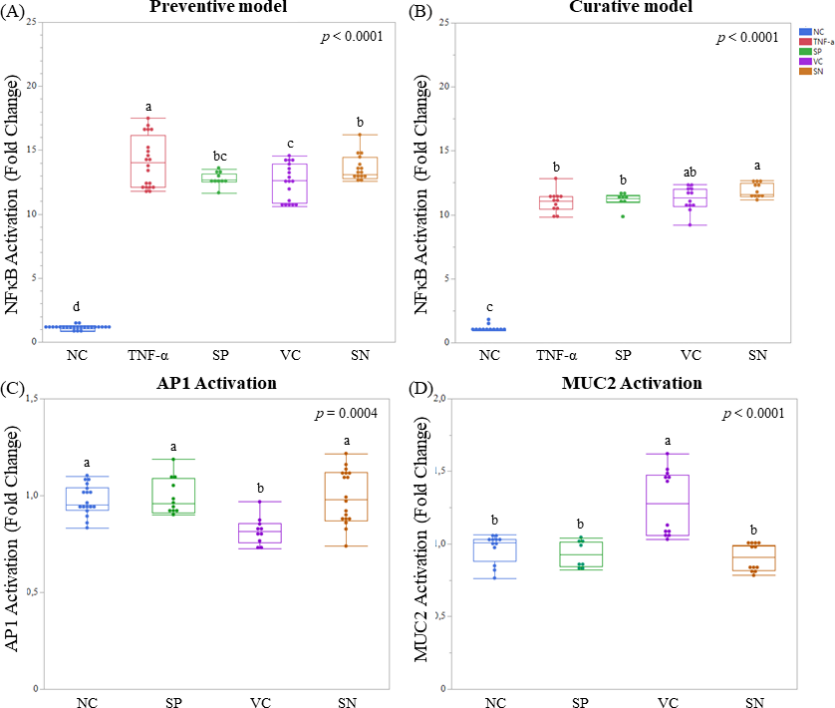
Effect of Bs29784 forms on HT-29-reporter cell lines regulation. Preventive model: pro-inflammatory stimuli with TNF- α 1 h after exposing HT-29-NF-κB reporter cell line to Bs29784 forms (**A**). Curative model: pro-inflammatory stimuli with TNF- α 1 h before exposing HT-29-NF-κB reporter cell line to Bs29784 forms (**B**). Impact of Bs29784 forms on AP-1 activity in HT-29-AP-1 reporter cell line (**C**). Impact of Bs29784 forms on MUC2 activity in HT-29-MUC2 reporter cell line (**D**). NC, negative control; SP, spores; VC, vegetative cells; SN, supernatant. The letters on the mustache representation show significant difference groups.

In the preventive model, both vegetative and spore forms of Bs29784 were able to significantly inhibit NF-κB activity induced by TNF-α in HT-29 cells. Vegetative cells showed the strongest anti-inflammatory effect decreasing the reporter expression by 14.8%, followed by spores, 12.2%. Interestingly, the supernatant of a culture of Bs29784 vegetative cells also reduced reporter gene expression but at a lesser extent (6.0%, *P* < 0.0001, Fig. 1A). In the curative model, none of the conditions tested counter the pro-inflammatory effect of TNF-α as observed by the sustained NF-κB expression (Fig. 1B). These results show that, in a cellular model, pre-exposition with Bs29784 spores, Bs29784 vegetative cells, or its metabolites sustains cellular resilience to pro-inflammatory challenges, as the one generated here by TNF-α, via the modulation of NF-κB expression. These results strengthen the widely accepted preventive, rather than curative, effect of probiotics. Furthermore, the slightly higher reduction of NF-κB activation by Bs29784 vegetative cells, when compared with its spores counterpart, suggests that this regulation requires, at least partially, the metabolic machinery of the probiotic.

To better characterize the anti-inflammatory effects of Bs29784 forms and its metabolites, we tested a complementary pathway to NF-κB by measuring the activation of the transcription factor AP-1 on HT-29 reporter cells. AP-1 activation is associated with a wide range of cellular processes including inflammatory disorders (20). In this model, only vegetative cells were able to significantly reduce AP-1 activation (16.1%, *P* = 0.0004, Fig. 1C). This suggests that the beneficial effect of Bs29784 may be linked with its metabolic activity, which, in turn, may reduce inflammation and maintain epithelium homeostasis.

### Bs29784 vegetative cells increase MUC2 activation

The mucus layer is the gut’s first line of defense, acting as a barrier against bacterial translocation and tissue damage. Mucus production is tightly regulated at the transcriptional level where MUC2 activation leads to mucin production, which is key to support gut barrier maintenance and integrity. In order to determine whether Bs29784 needed to be metabolically active to stimulate mucus production via MUC2 activation, we assessed MUC2 expression upon exposure with Bs29784 spores, vegetative cells, and its metabolites using the same type of reporter system described above. After 24 hours of incubation, only Bs29784 vegetative cells were able to significantly activate MUC2 in our reporter system. This activation exceeded the negative control by 32.2% (*P* < 0.0001, Fig. 1D). This result suggests that Bs29784 may require to be metabolically active to promote activation of MUC2, probably via its metabolites, which, in consequence, may lead to epithelial protection and strengthening of the intestinal barrier.

### Bs29784 metabolites HPX, NIA, and PTH differentially regulate global transcription factors and, thus, intestinal health

Above, we showed that Bs29784 vegetative cells exert a higher anti-inflammatory activity and epithelial protection via NF-κB and AP-1 inhibition and MUC2 activation, respectively, than their spore’s counterpart. Previously, we showed that probiotic Bs29784 releases a large panel of metabolites with a variate biological function both *in vitro* and *in vivo* (chicken gut) (14). Because of their biological function and statistical significance, among the metabolites identified, the more relevant were HPX, NIA, and PTH (14). Accordingly, we hypothesized that the anti-inflammatory activity and epithelial protection of Bs29784 vegetative cells described above are mediated by these metabolites. To test this hypothesis, we tested chemically pure forms of HPX, NIA, and PTH on the HT-29 reporter systems as a way to determine their individual contribution to these phenotypes.

**Fig 2:**
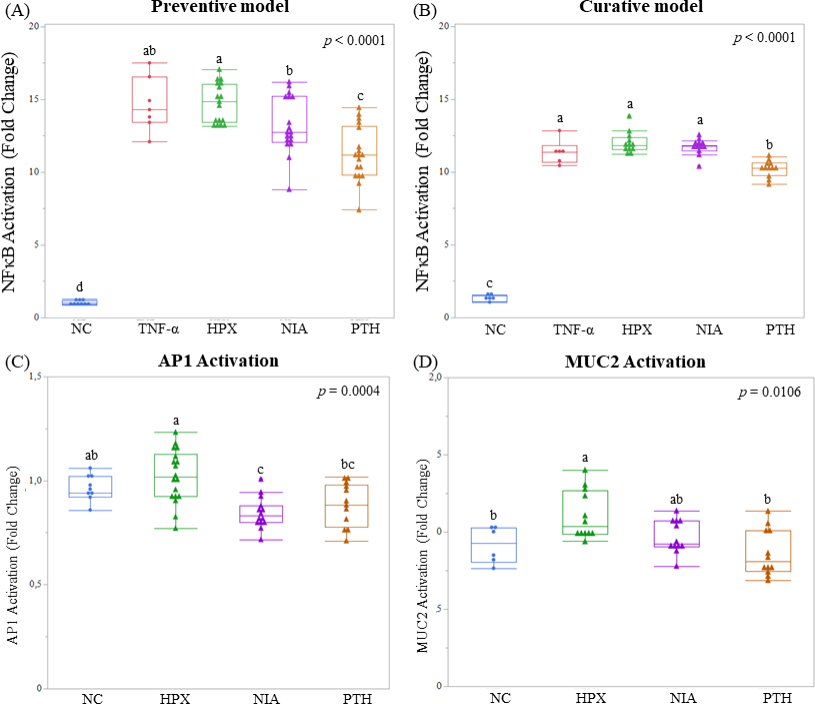
Effect of Bs29784 metabolites: HPX, NIA, and PTH on HT-29-reporter cell lines regulation. Preventive model: pro-inflammatory stimuli with TNF- α 1 h after exposing HT-29-NF-κB reporter cell line to metabolites (**A**). Curative model: pro-inflammatory stimuli with TNF- α 1 h before exposing HT-29-NF-κB reporter cell line to metabolites (**B**). Impact of Bs29784 forms on AP-1 activity in HT-29-AP-1 reporter cell line (**C**). Impact of Bs29784 forms on MUC2 activity in HT-29-MUC2 reporter cell line (**D**). NC, negative control; HPX, hypoxanthine; NIA, niacin; PTH, pantothenate. The letters on the mustache representation show significant difference groups.

Incubation with Bs29784 metabolites, HPX, NIA, and PTH, with the HT-29-NF-κB reporter system differentially regulated NF-κB inhibition upon the inflammatory challenge (TNF-α). Indeed, PTH was the only metabolite to significantly decrease NF-κB activation in both the preventive and curative model (23.2% and 10.2%, respectively; *P* < 0.0001) (Fig. 2A and B). This effect was only partially observed in the preventive model with NIA (9.7%; Fig. 2A). Whereas, HPX did not show any positive effect on NF-κB pathway in either the preventive or curative model. Regarding the complementary immunomodulatory AP-1 pathway, its activation was significantly decreased by NIA by 12.1% and numerically by 7.8% by PTH (*P* = 0.0004) (Fig. 2C). Finally, on the HT-29-MUC2 reporter system, only HPX shows a substantial effect by activating MUC2 expression by 19.3% (Fig. 2D) (*P* = 0.0106).

Altogether, these results suggest that Bs29784 metabolites HPX, NIA, and PTH differentially regulate global transcription factors and, thus, intestinal health. Indeed, the immunomodulatory properties of Bs29784 vegetative cells via NF-κB and AP-1 inhibition are partially mediated by its secreted metabolites NIA and PTH, whereas intestinal protection via MUC2 activation and, thus, mucus production is principally mediated by HPX.

### HPX, PTH, and NIA promote enterocyte proliferation and protect them from an pro-inflammatory stimuli

Inhibition or activation of global transcription factors leads to the regulation of complex signaling pathways with variate downstream effects. In the case of NF-κB and AP-1, their inhibition can lead to the reduction of the secretion of pro-inflammatory cytokines (21). Concomitantly, AP-1 modulation and MUC2 activation stimulate epithelial protection and homeostasis by strengthening the epithelial barrier (22).

To further characterize the biological effect of Bs29784 metabolites HPX, NIA, and PTH and their downstream effects upon modulation of global transcription factors, we assessed their individual effect on cell proliferation, transepithelial electrical resistance (TEER), and response to pro-inflammatory challenge on Caco-2 cells.

**Fig 3:**
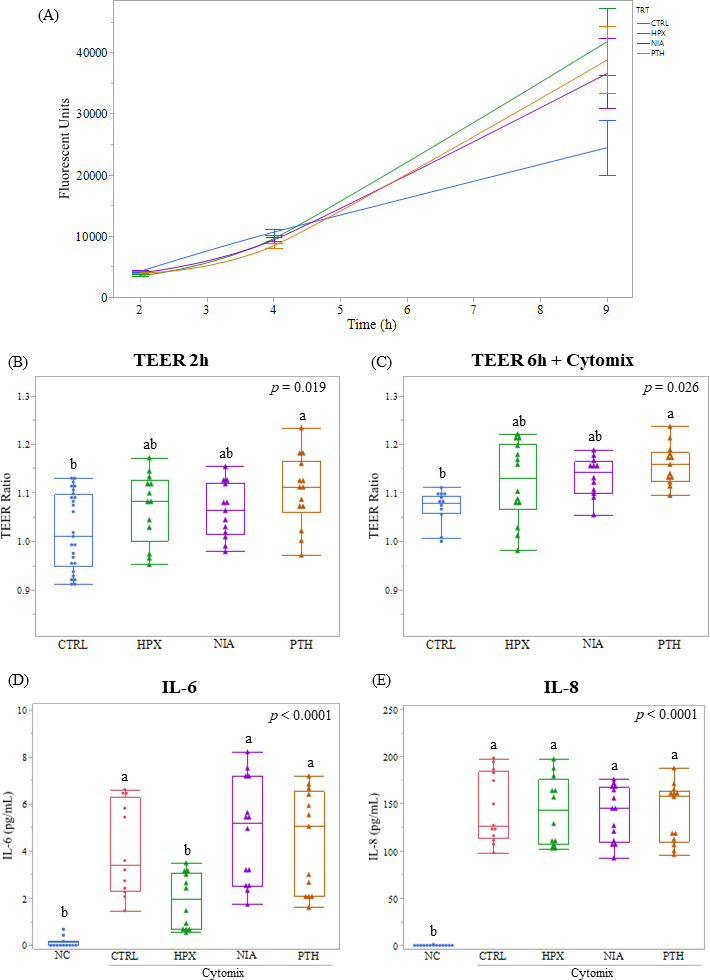
Impact of HPX, NIA, and PTH on *in vitro* intestinal resilience. (**A**) Effect of metabolites on Caco-2 cell proliferation (**B**) basal TEER measurements after a 2-h exposure with the tested metabolites. (**C**) TEER measurements after a 6-h exposure with the tested metabolites and Cytomix supplementation; IL-6 (**D**) and IL-8 (**E**) quantification upon a pro-inflammatory challenge.

Regarding cell proliferation, we used the PrestoBlue assay, which is a cell viability indicator that shows a good correlation between cell number and the reducing power of living cells (23). From day 0 to day 4, no significant difference in fluorescence signal was observed compared with the control (Fig. 3A). However, on day 9 post-seeding, in the presence of 1 mM of HPX, PTH, or NIA, the fluorescent signal increased by 73.5% (*P* = 0.011), 59.8% (*P* = 0.012), and 50.4% (*P* = 0.059), respectively. This indicates that Bs29784 metabolites HPX, NIA, and PTH increase cell proliferation *in vitro*.

In order to assess the effect of Bs29784 metabolites HPX, NIA, and PTH on cellular resilience to an inflammatory challenge, we exposed Caco-2 cells to a pro-inflammatory cytokine cocktail containing IL-1β, INF-γ, TNF-α (CytoMix), and assessed their response by measuring TEER and cytokine production in the presence or absence of our metabolites of interest. Caco-2 cell stimulation with the CytoMix in the absence of Bs29784 metabolites decreased TEER values by 27% and increased the release of pro-inflammatory chemokines IL-6 (10-fold) and IL-8 (340-fold). Decreased TEER values reflect an increase in epithelial permeability, which *in vivo* translates on gut leakage from lumen to the lamina propria and systemic circulation. When treated with PTH, Caco-2 cells increased TEER values in the absence of pro-inflammatory stimuli (Fig. 3B). Furthermore, PTH partially protected epithelial integrity from CytoMix by limiting the reduction of TEER upon challenge (Fig. 3C). HPX, on the other hand, exerts its function by reducing IL-6 levels upon acute pro-inflammatory stimuli (50% decrease) (Fig. 3D). IL-8 secretion was not significantly impacted by any of the Bs29784 metabolites tested (Fig. 3E). Overall, the Caco-2 cellular model showed that Bs29784 metabolites have different modes of action on cell proliferation and intestinal resilience.

### NIA, HPX, and PTH modify ileal and cecal SCFA production and microbiota composition

Gut microbiota is an important pillar of animal resilience and intestinal health as it provides a protective barrier against infection and plays a key role in promoting and maintaining intestinal immune homeostasis. Probiotic Bs29784 has been previously reported to modulate gut microbiota by increasing its diversity and stimulating short-chain fatty acid (SCFA) producers (9, 10). However, the exact mechanism by which these changes occur is still unknown. In consequence, we hypothesized that microbiota modulation by Bs29784 maybe mediated by its secreted metabolites HPX, NIA, and PTH. To address this question, we performed an *in vitro* fermentation assay to assess the influence of the Bs29784 metabolites on SCFA profile and microbiota composition.

NIA and PTH favored *in vitro* fermentation by increasing total gas production in the caecum by 8% and 11.4% (*P* = 0.011), respectively, and decreasing pH in the ileum in 0.1 units (*P* = 0.043). In addition, HPX and PTH were able to decrease the lag time in the ileal fermentation by 4% and 5.3% (*P* = 0.007) (Table 1). This means that the microbiota adapted quickly to the new environment and fermentation might begin sooner. The addition of PTH to the ileal fermentation decreased acetate production (*P* = 0.012) and tended to increase butyrate production (*P* = 0.076). In the cecal fermentation, NIA generated a significant increase in propionate production by 6.7% (*P* = 0.016), whereas HPX and PTH increased butyrate production by 5.7% and 6.8 % (*P* = 0.019), respectively, and tended to increase overall SCFA production (*P* = 0.087). The addition of those molecules did not affect protein fermentation in any of the compartments (Table 1).

**TABLE 1:** Cecal and ileal microbial metabolites and fermentation parameters^*a*^

Origin	Parameters		CTRL^*b*^	HPX	NIA	PTH	P-value
Ileum	Microbial metabolites (mM)	Acetate	12.49^*a*^	13.34^*a*^	11.9^*a*^	9.82^*b*^	0.012
		Butyrate	0	0	0	0.31	0.076
		SCFAs	12.49^*a*^	13.34^*a*^	11.9^*ab*^	10.24^*b*^	0.017
		NH_3_	4.87	5.27	4.47	4.89	0.37
		Lactate	5.05	6.95	9.15	7.78	0.131
	Fermentation parameters	Lag (min)	293.92^*a*^	282.15^*bc*^	287.82^*ab*^	278.36^*c*^	0.007
		Slope (mba/min)	0.3	0.31	0.33	0.32	0.626
		Max pressure (mba)	360.8	374.83	395.75	391.48	0.539
		pH	6.8^*a*^	6.75^*ab*^	6.69^*b*^	6.72^*b*^	0.043
Caecum	Microbial metabolites (mM)	Acetate	43.11	43.4	45.31	45.52	0.112
		Propionate	17.78^*bc*^	17.14^*c*^	18.97^*a*^	18.67^*ab*^	0.016
		Butyrate	7.6^*b*^	8.03^*a*^	7.8^*ab*^	8.12^*a*^	0.019
		Valerate	0.22	0.22	0.22	0.22	1
		Isobutyrate	1.11	1.08	1.08	1.08	0.894
		SCFAs	68.72	68.8	72.3	72.03	0.087
		NH_3_	10.13	10.07	9.5	9.43	0.836
		Lactate	0.004	0.0001	0	0.0111	0.067
	Fermentation parameters	Lag (min)	0	0	0	0	1
		Slope (mba/min)	0.23	0.23	0.23	0.24	0.744
		Max pressure (mba)	475.25^*b*^	503^*a,b*^	513.25^*a*^	529.25^*a*^	0.011
		pH	6.71	6.75	6.7	6.71	0.328

^*a*^ Mean values with different superscripts in a row differ significantly.

^*b*^ CTRL, control; HPX, Hypoxanthine; NIA, niacin; PTH, pantothenate.

The metabolic changes identified were the result of microbiota modification and fermentation that occurred over 24 hours. A 16S rRNA study was performed to examine microbiota shift caused by HPX, NIA, and PTH. The next-generation sequencing (NGS) data set generated a total of 13,500 sequences per sample after normalization for a total of 216,000 sequences. The chao index revealed that for the caecum, there were between 26% and 30% of unseen species and less than 10% for the ileum, confirming that the sequencing depth was enough to have a good coverage of the microbial diversity.

The microbial richness in the microbiota assay was maintained at a high level in the caecum with 130 to 140 amplicon sequence variants (ASVs) detected on average, whereas it was much lower in the ileum with only 10 to 17 ASVs detected. A tendency to increase diversity (Simpson and Shannon index) was observed in the caecum for all three molecules (Table 2).

**TABLE 2:** Cecal and ileal microbial diversity and richness

Origin	Parameter	CTRL^*a*^	HPX	NIA	PTH	P-value
Caecum	Richness	131.5	139.5	141	141.3	0.69
	Chao	170.8	164.5	201.7	234	0.43
	Simpson	0.51	0.59	0.57	0.58	0.09
	Shannon	1.82	2.11	2.01	2.05	0.12
Ileum	Richness	13	11.3	10	17	0.23
	Chao	14	11.3	10.8	17.5	0.25
	Simpson	0.55	0.53	0.57	0.56	0.11
	Shannon	0.98	0.93	0.99	1.04	0.14

^*a*^ CTRL, control; HPX, Hypoxanthine; NIA, niacin; PTH, pantothenate.

**Fig 4:**
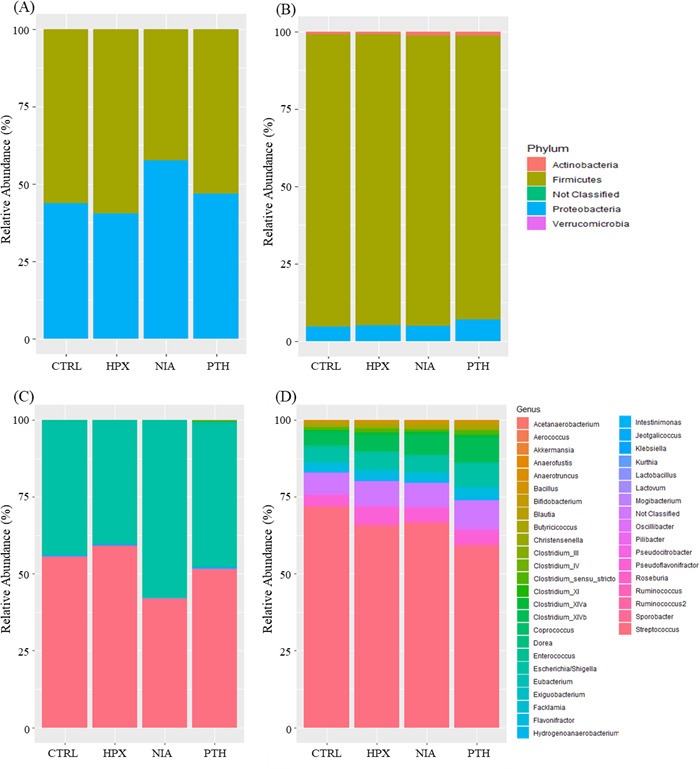
Impact of Bs29784 metabolites on microbiota composition at phylum (**A, B**) and genera taxonomic levels (**C, D**) in ileal (**A, C**) and cecal fermentations (**B, D**).

In the ileum, the microbial population shift caused by Bs29784 metabolites occurred mainly at the phylum level, with only NIA and PTH influencing ileal microbiota (Fig. 4A). HPX had no effect on the ileal microbiome. PTH favored the development of *Actinobacteria* (*P* adj. = 0.04), whereas NIA favored a shift significantly increasing *Proteobacteria* while decreasing *Firmicutes* (*P* adj. = 0.04). At the genus level, PTH enhanced by sixfold the abundances of *Bifidobacterium* (*P* adj. = 0.10) and the emergence of *Facklamia* in the ileal microbial profile (*P* adj. <0.001) (Fig. 4C).

The cecal microbiota was modified by all three Bs29784 metabolites. At the phylum level, NIA significantly increased *Actinobacteria* proportion (*P* adj. = 0.03) (Fig. 4B). At the genus level, NIA considerably increased the abundances of *Bifidobacterium* (*P* adj. = 0.04) and SCFA producers such as *Ruminococcus* (*P* adj. = 0.01), *Clostridium*_XIVb (*P* adj. = 0.04), and *Anaerotruncus* (*P* adj. = 0.04) (Fig. 4D). HPX promoted the development of members of the *Oscillospiraceae* family including *Ruminococcus* (*P* adj. = 0.01), *Anaerotruncus* (*P* adj. = 0.04), and *Pseudoflavonifractor* (*P* adj. = 0.04) populations at expenses of *Bifidobacterium* abundances. Finally, PTH doubled *Ruminococcus* population (*P* adj. = 0.01) and *Clostridium*_XIVb (*P* adj. = 0.04) (Fig. 4D).

**Fig 5:**
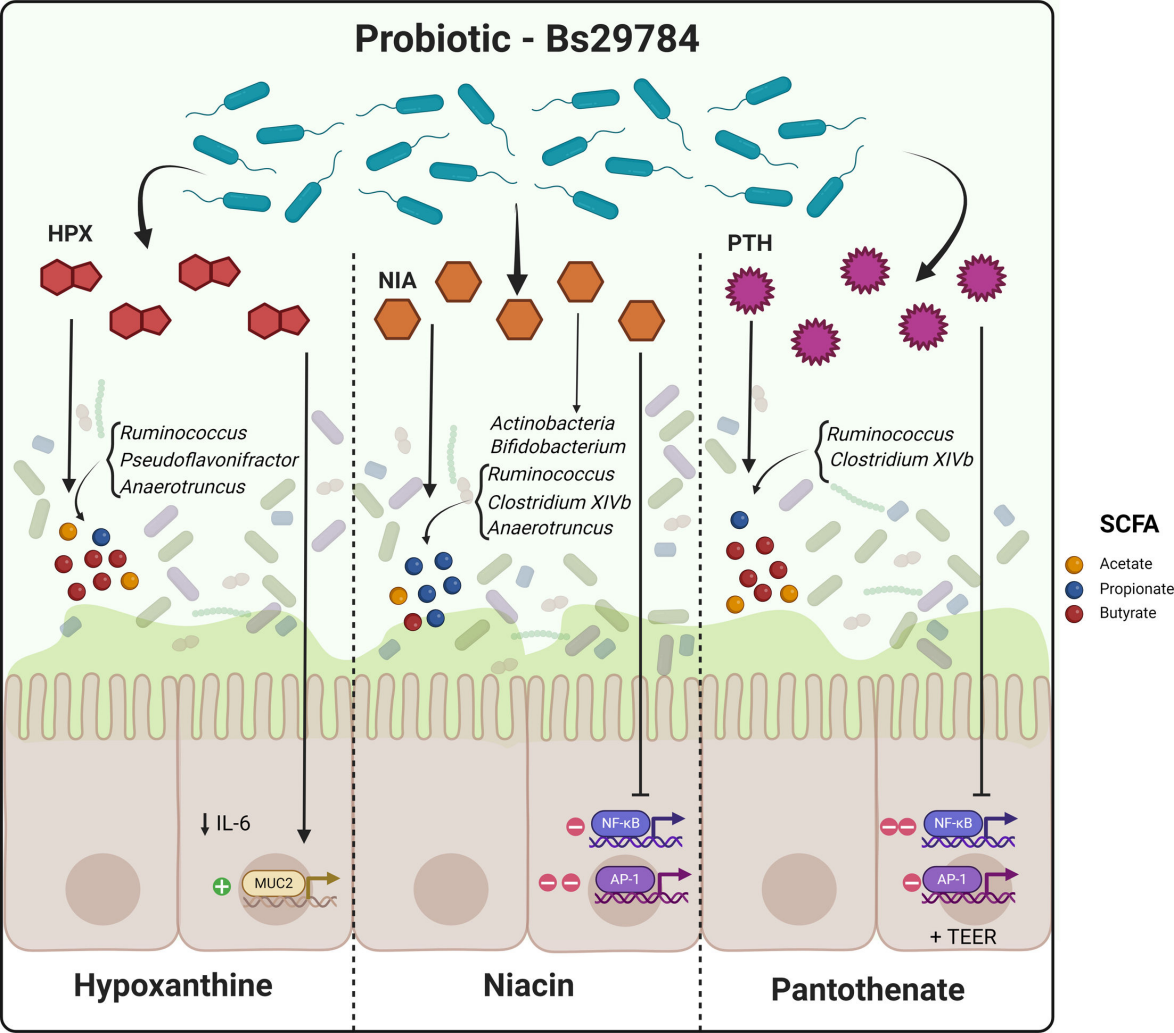
Overview of Bs29784 metabolites effects on intestinal immunomodulation, gut integrity, and modulation of complex gut microbial ecosystem. Created with BioRender.com.

Overall, Bs29784 metabolites at 1 mM shifted a complex gut microbial population in various ways. Their effect was more pronounced in the cecal fermentation than in the ileal fermentation. They were able to boost fermentation capacity, change metabolite profiles, and modify microbiota composition at the phylum and genus levels without major alterations on diversity (Fig. 5).

## DISCUSSION

After the ban of antibiotics as growth promoter in many countries, animal feed industries have developed numerous possible alternatives, among them probiotics (24). Probiotics are defined as live microorganisms, which, when administered in adequate amounts, confer a health benefit on the host (25). They are known to offer health benefits through their antimicrobial activity against pathogens through competitive exclusion of adhesion sites and nutrients, generation of bacteriocins, and anti-inflammatory activities that modulate the immune system. They have the ability to modify microbiota, maintain and strengthen gut barrier function, and promote adequate nutrient absorption (26, 27). Nevertheless, including them in animal feed is difficult because their action is dependent on their survival after pelleting and passage through the GIT. To overcome these constraints, the livestock industry has successfully implemented the use of spore-forming probiotic microorganisms. Hence, one of the most widely used genera in animal nutrition is *Bacillus* (28, 29). They are able to sporulate and, thus, withstand pelleting and GIT conditions. Once in the intestine, they germinate and become metabolically active, allowing them to synthesize short chain fatty acids, secrete antibacterial compounds and enzymes, and compete for essential resources with pathogens, all of which benefit the host (30, 31). In order to gain deeper knowledge of how spore-forming probiotics improve gut health, we focused our work on two main points: first, the characterization and comparison of the two metabolic states of the spore-forming probiotic, Bs29784, and second, the characterization *in vitro* of the health benefits of the metabolites produced by Bs29784. For the latter, we used chemically pure forms of the metabolites NIA, PTH, and HPX because we did not observe any significant impact of the supernatants of vegetative cells on global transcription factor expression. The lack of response using supernatants generated *in vitro* can be explained by the technical limitation of supplying metabolites to the cells in an unknown concentration or proportion.

One of the three animal resilience’s pillars is the immune system. Here, we showed that Bs29784 spores exert an anti-inflammatory effect in a preventive model when cells were challenged with TNF-α. Although, the greatest effect was observed with vegetative cells. Furthermore, the spores’ mode of action was accounted by reducing NF-κB activation rather than AP-1. This effect can be explained by the spore crust, which contains numerous enzymes and proteins (32, 33). However, not all studies on spores show an anti-inflammatory effect; Duc and colleagues (34) described an increase of pro-inflammatory cytokines when spores of *Bacillus pumilus* and *B. subtilis* were in contact with a cultured macrophage cell line. Huang and colleagues (35) demonstrate that spores failing to germinate were not likely to interact with toll-like receptors (TLR), particularly TLR2 and TLR4, but their vegetative cells do. In our model, even if an anti-inflammatory effect was observed with Bs29784 spores, the vegetative cells had the strongest preventive effect against inflammation by reducing the activation of two different immune pathways: NF-κB and AP-1. It has been reported that the anti-inflammatory capacity of vegetative cells is based on their ability to modify the cross-talk between gut microbiota and gut immune system by the transcription factor NF-κB (36). Previously, we studied other pathways related with the anti-inflammatory potential of Bs29784 (12). In that work, upon exposure to Bs29784 vegetative cells, we showed a significant decrease in IL-8 production in stress conditions, stimulated by pro-inflammatory IL-1β, and reduced upregulation of iNOS levels. Here, we were unable to reproduce this effect using only the individual purified forms of Bs29784 metabolites at 1 mM. This suggests that IL-8 reduction by Bs29784 requires a more complex set of metabolites produced by its vegetative cells as previously reported by other sporulated bacteria (14, 37). Altogether, Bs29784 anti-inflammatory activity is, therefore, explained by their cell wall structures and metabolic activity. We further investigated the role of the previously identified major metabolites produced by Bs29784 in the GIT of broilers supplemented with this probiotic, named NIA, PTH, and HPX (14). When these molecules were tested *in vitro*, we observed a significant anti-inflammatory effect of NIA and PTH in a preventive model, with PTH having the strongest effect. PTH is the only metabolite that significantly reduces NF-κB activation in a curative model. Both NIA and PTH are vitamins and precursors of key molecules for intestinal homeostasis and energy metabolism such as nicotinamide adenine dinucleotide (NAD) and Coenzyme A (CoA), respectively (38, 39). Our findings are consistent with the literature, which indicates that NIA presents anti-inflammatory and antioxidative capabilities modulating the activity of various transcription factors such as the inhibition of NF-κB, stimulation of SIRT_1_ (40), or mediating anti-inflammatory response on activated macrophages (38, 41). Furthermore, NIA reduces inflammation in a Caco-2 cell model upon challenge with IL-1β or lipopolysaccharide (LPS), probably through modulation of the NAD precursor status and the significant shift in metabolism to boost immune response via lipid mediators and glycolysis LPS (42). Although PTH’s anti-inflammatory effects have been less studied (43), it has been shown to regulate inflammatory response by repressing Th17 cell differentiation (44). Concerning HPX, a recent study suggested it might inhibit pro-inflammatory secretion of IL-1 by LPS- and IFN-stimulated macrophages (45). This finding is in line with ours. Indeed, we also observed a decrease of pro-inflammatory IL-6 production by intestinal cells following an acute stimulation with CytoMix. However, this effect seems to be mediated by other pathways than NF-κB and AP-1, as suggested by the lack of signal in our HT-29 reporter cells model (Fig. 2).

The second pillar of animal resilience, gut integrity, is based on the maintenance of a strong epithelial layer (high *in vitro* TEER values of cell monolayers) protected *in vivo* by a mucus layer. Here, we assessed this pillar by measuring three parameters: epithelial proliferation, TEER upon pro-inflammatory challenge, and MUC2 expression in the HT-29 reporter system. We showed that Bs29784 vegetative cells were able to activate MUC2 (Fig. 1D) and, thus, mucus production probably via the secretion of HPX (Fig. 2D). This activation would translate *in vivo* to an improvement of intestinal barrier function. This capacity was already shown *in vitro* on other vegetative cells of *Bacillus* spp. (46) and other kinds of probiotics such as *Lactobacillus* (47). These results are in accordance with Lee and colleagues (16) who showed that HPX is essential for the proper functioning of the intestinal barrier due to the regulation of energy metabolism in intestinal epithelial cells. Moreover, we showed that the three Bs29784 metabolites tested increased Caco-2 cells’ proliferation (Fig. 3A). Previously, it has been demonstrated *in vitro* that PTH increased wound healing in human fibroblast (48); nicotinamide, an amide form of NIA, promoted cell survival and differentiation in human pluripotent stem cells (49). Moreover, Lee and colleagues (16) demonstrated the significant increase of wound closure on T84 cell lines upon exposure with HPX, concluding that HPX plays a key role on intestinal barrier by modulating energy metabolism. Finally, we showed that PTH enhanced intestinal resilience to an inflammatory challenge by limiting TEER reduction (Fig. 3C). These results highlight the importance of PTH to maintain intestinal integrity and are in line with previous research *in vivo* showing that its deficiency in animals could lead to mild to severe ulcerative colitis (50).

Finally, we assessed the third pillar of animal resilience, the microbiota. Gut microbiota is an important barrier against pathogens. It produces metabolites, which are energy sources to enterocytes and provides micronutrients required for the proper functioning of the epithelium (51, 52). Here, we showed that supplementation of Bs29784 metabolites modulates both ileal and cecal microbial fermentations. These metabolites may play an important role through cross-feeding because 20%–30% of the gut microbiota does not synthesize essential B vitamins such as NIA and PTH (53). PTH was the only metabolite tested able to modify the SCFA profile in the ileal fermentation by decreasing acetate levels and increasing butyrate. The cecal butyrate concentrations were also increased. Furthermore, we reported that PTH supplementation increased *Actinobacteria* populations; those results are in line with Magnúsdóttir and colleagues (54), where the addition of PTH favors *Actinobacteria* due to their lack of PTH biosynthesis pathway. The increase of *Actinobacteria*, especially *Bifidobacterium*, favors butyrate-producing bacteria, as there is a cross-feeding bacterial interaction (55). This report supports our findings of increased butyrate concentration in ileal and cecal fermentations (Table 1). Regarding NIA, we found a significant increase mainly in *Lachnospiraceae* family represented by *Ruminoccocus*, *Clostridium*_XIVb, and *Anaerotruncus*, which are members of a major butyrate-producing family (56). Our results are consistent with those of Liu and colleagues (57), who also detected an increase of *Lactobacillus* and *Dorea* abundance when weaned piglets were supplemented with NIA. In this study, it was observed that HPX, a co-metabolite produced by both microbial and host metabolism (58), increased *Oscillospiraceae* population particularly *Ruminococcus.* The same strong correlation was described by Wakita and colleagues (59), suggesting that the HPX concentration depends on the numbers of these bacteria. Based on this study and our data, we hypothesize that HPX is a cause for *Ruminococcus* increase rather than a consequence.

Altogether, with this work, we confirmed the anti-inflammatory activity of Bs29784 spores, vegetative cells, and the most represented metabolites of Bs29784. We deepened in the understanding of the individual activities of each of these metabolites on the three pillars of animal resilience: intestinal immunomodulation, gut integrity, and modulation of complex gut microbial ecosystem (Fig. 5). Our findings suggest that Bs29784 and its metabolites play a key role in maintaining gut homeostasis through both direct and indirect mechanisms, and explain partly its mode of action on host health, resilience, and performance (9–11). Further work remains to be completed in order to determine the full spectrum of activities of Bs29784 metabolome both *in vivo* and *in vitro*.

## MATERIALS AND METHODS

### Bs29784 culture conditions

The bacterial strain used in the study is commercially available and used as probiotic in animal feed. Bs29784 was cultured overnight on Tryptone Soya Broth (TSB) at 37°C. Optical density (OD) at 600 nm was measured to verify that culture reached 10^9^ CFU/mL. The resulting culture was centrifugated at 10,000 × *g* for 10 min at 4°C. Pellet was separated from the supernatant and resuspended in 1× phosphate-buffered saline (PBS). Concerning the spores, Bs29784 spores powder was resuspended in PBS to a concentration of 10^9^ CFU/mL. For HT-29 reporter cell lines study, the resuspended pellet, supernatant, and spores were diluted to a final concentration of 10^7^ CFU/mL.

### Regulation of inflammatory response and mucin production by reporter cell lines

The human intestinal epithelial cell line HT-29 was obtained from the American Type Culture Collection (ATCC, Rockville, MD, USA), cultured at 37°C with 5% CO_2_ in RPMI 1640 medium (Sigma) supplemented with 10% heat-inactivated fetal bovine serum (FBS, Eurobio), 50 U/mL penicillin, and 50 U/mL streptomycin. The reporter cell lines were established as follows:

HT-29-NF-κB reporter cell line: the reporter plasmid pNiFty2SEAP (Invivogen) containing NF-κB response elements in its promoter was used to establish a stable reporter cell line after selection by antibiotic (zeocin, 50 µg/mL, Invivogen) and was selected for its response to 10 ng/mL TNF-α after 24 hours of stimulation, as described by Lakhdari and colleagues (17). For the experiments, HT-29-NF-κB cells were seeded at 5 × 10^4^ cells/well of 96-well plate and were incubated for 48 hours before adding the treatments (Bs29784 spores, vegetative cells and its supernatant, HPX, NIA, and PTH; Sigma). HPX, NIA, and PTH were supplemented at a final concentration of 1 mM per well, which is an intermediate value of the range (0.1–2.44 mM) previously reported to be active in other cellular models (38, 41, 42).

HT-29-AP-1 reporter cell line: the reporter plasmid pAP-1-Luc (containing seven AP-1 binding site cloned in the pcDNA3.1-Luciferase plasmid) was used to establish a stable HT-29-AP-1 reporter cell line after antibiotic selection (zeocin, 50 µg/mL, Invivogen) and was selected for its response to PMA 0.1 µM after 24 hours of stimulation. For the experiments, HT-29-AP-1 cells were seeded at 3 × 10^4^ cells/well and were incubated for 24 hours before adding the treatments (60).

HT-29-MUC2 reporter cell line: the reporter plasmid pGL4.14-MUC2-Luc was used to establish a stable HT-29-MUC2 reporter cell line after antibiotic selection (hygromycin, 600 µg/mL, Invivogen) and was selected for its response to 10 ng/mL TNF-α after 24 hours of stimulation. For the experiments, HT-29-MUC2 cells were seeded at 5 × 10^4^ cells/well and were incubated for 48 hours before adding the treatments.

For each experiment, cells were seeded at the appropriate density and were incubated for the required time at 37°C in 5% CO_2_ atmosphere, depending on the cell line. The cells were then stimulated with 10 µL of samples (10% vol/vol) and were incubated for 24 hours prior to the quantification of the reporter gene activation (SEAP or luciferase). For the activated conditions, the corresponding activators were added either 1 hour before (curative model) or after (preventive model) the addition of the samples. SEAP or intracellular luciferase activity was revealed using QUANTI-Blue (Invivogen) and Neolite (PerkinElmer) reagents, respectively. SEAP activity at 655 nm optical density (OD_655_) and luciferase activity expressed in relative light unit (RLU) were measured using Synergy 2 microplate reader (BioTeck). To assess the effect of each sample on cellular viability, mitochondrial activity was quantified using MTS assay (Promega).

### Proliferation assay

The cells (Caco-2, DSMZ, Deutsche SammLung von Mikroagencen und Zellkulturen GmbH, ACC 169) were cultured in a T75 flask at 37°C/5% CO_2_ in complete Dulbecco's modified Eagle medium (DMEM) (10% FBS, 1% non-essential amino acids [NEAA], 1% sodium pyruvate, and 2 mM L-glu; Thermo Fisher) and were used between passages 5 and 25. The medium was changed three times per week, and the cells were passaged every week (80%–90% confluent, trypsinized, counted on Countess II, and reseeded at 180,000 cells/flask).

The 96-well plates (Thermo Fisher) were inoculated with 7,000 cells/well and were cultured for 9 days (three changes of medium per week: 250 µL/well) in complete DMEM supplemented with PTH, NIA, or HPX at 1 mM at 37°C/5% CO_2_. The proliferation of Caco-2 cells was based on the measurements of their metabolic activity using “PrestoBlue.” PrestoBlue Cell Viability Reagent was a ready-to-use, non-toxic, resazurin-based solution that functions as a cell health indicator by using the reducing power of living cells to quantitatively measure viability. Briefly, at day 2, 4, 7, and 9, the medium was replaced by 250 µL of PrestoBlue solution in DMEM and was incubated for 1 hour at 37°C, 5% CO_2_. Following the incubation, the fluorescence was measured—the excitation range was 540 nm and an emission of 590 nm. The signal intensity is proportional to the number of cells, and the results were expressed in fluorescent units.

### Resilience to inflammation assay

The 12-well insert plates (Costar, 1 cm^2^) were seeded at 100,000 cells/well and were cultured for 15 days (three changes of medium per week: 500 µL apically and 1.5 mL basally) in complete DMEM. PTH, NIA, and HPX were prepared at 100 mM in PBS, NaOH 100 mM, and water, respectively, filtered sterilized, and then diluted 1/100 in complete DMEM (1 mM final concentration). The cellular assay was performed on day 16 post-seeding. The TEER was measured (must be between 700 and 800 Ω/cm^2^, Evom, WPI) at T 0h. The treatments were then applied in the apical compartment. The cells were incubated for 2 hours at 37°C/5% CO_2_, the TEER was measured, then 150 µL of DMEM was taken basolaterally and was replaced by 150 µL of 10× CytoMix. The CytoMix 1× contains 1 ng/mL of IL1, 10 ng/mL of TNF-α, and 5 ng/mL of IFN (Sigma). The cells were incubated for another 4 hours at 37°C/5% CO_2_. The TEER was measured, and the basolateral and apical fractions were taken. Results were expressed in megaohms per centimeter. The samples were stored at −80°C, and the IL-8 and IL-6 were measured by enzyme-linked immunosorbent assay (ELISA) (Thermo Fisher); results were expressed in picograms per milliliter.

### Modulation of gut microbiota

All experiments were conducted according to the European Union Guidelines of Animal Care and legislation governing the ethical treatment of animals, and investigators were certified by the French government to conduct animal experiments. The Center for Expertise and Research in Nutrition facilities (Malicorne, France) are in accordance with the agreement no. C 03 159 4 of 6 November 2008, relative to experimentation on vertebrate living animals (European regulation 24/11/86 86/609 CEE; Ministerial decree of 19 April 1988). Twelve 28-day-old broilers chickens were euthanized, and their cecal and ileal contents were collected, pooled, and immediately stored at −80°C.

Cecal and ileal chicken microbial fermentation was performed in Hungate tubes in anaerobic buffer prepared according to Davies and colleagues (61). Four technical replicates were performed by condition. The anaerobic buffer was composed of five solutions (A, B, C, D, and E) prepared individually: solution A (per liter: 5.7 g Na_2_HPO_4_, 6.2 g KH_2_PO_4_, and 0.6 g MgSO_4_-7H_2_O), solution B (per liter: 4 g NH_4_HCO_3_ and 35 g NaHCO_3_), solution C (per 10 mL: 132 mg CaCl_2_-2H_2_O, 100 mg MnCl_2_-4H_2_O, and 80 mg FeCl_3_-6H_2_O), solution D (per liter: 0.1% resazurin), and solution E (per 100 mL: 4 mL de NaOH 1M, 625 mg de Na_2_S). The anaerobic buffer was assembled in anaerobic condition the day of the experiment (gazed with a mixture of CO_2_ and N_2_): 0.01% of solution A, 25.3% of solution B, 25.3% of solution C, 0.1% of solution C, 49.29% of ultra-pure water (18.2 mΩ) and autoclaved in the presence of 0.5 g/L of L-cysteine (reducing agent). On the day of the experiment, the buffer was further reduced by adding 4% (vol*/*vol) of solution E before adding the cecal inoculum (final pH: 7.5 and Eh: −150 mV). The cecal content was mixed at 5% (wt*/*vol) with the anaerobic buffer, and 10 mL of the slurry was transferred in Hungate tubes. All treatments were added at 1 mM. The fermentation was then performed in water bath under constant agitation (200 rpm) at 39°C during 24 h. During the fermentation, the gas pressure was recorded at T0, T2h, T4h, T6h, T8h, and T24h by means of pressure transducer (Comark C9551) connected to a 21-g needle. At the end of the fermentation, the pH was recorded, and samples were collected to quantify SCFAs (quantification performed by gas chromatography Agilent 6890 with 2-ethyl-butyric acid as internal standard, and acetonitrile for solvents equipped with FID detector and ZB-FFAP column) and lactate (Megazyme, K-DLATE), NH3 (Megazyme, K-AMIAR), and microbiota composition (Illumina sequencing, Genoscreen, France). From the gas production curve (fitted using polynomial regression), lag time, slope, and the total gas pressure were calculated and expressed, respectively, in minutes, millibar per hour, and millibar.

Sequencing of 16S was carried out on Illumina platform at GenoScreen using the Metabiote kit on the V3-V4 16S hypervariable region. Briefly, DNA was extracted and normalized, and the multiplex library (30 samples using unique indexes) was prepared for Illumina Miseq paired-end 2 × 300 bases. Quality control of the sequencing was performed using a mock community (15 bacterial and 2 archaeal strains) including the sequencing run. The primer and index were identified (100% homology) and removed to create demultiplexed fastq files. The fastq files were quality trimmed at Q30 at the end of the read, the reads were then paired assembled with minimum 30-bp alignment at 97% homology using Qiime. The demultiplexed, quality-trimmed, and assembled reads were then clustered using DADA2 software. The DADA2 package infers exact ASVs from high-throughput amplicon sequencing data, replacing the coarser and less accurate operational taxonomic unit (OTU) clustering approach. The DADA2 pipeline takes demultiplexed fastq files as input and outputs the sequence variants and their sample-wise abundances after removing substitution and chimera errors. Taxonomic classification is carried out via a native implementation of the RDP naive Bayesian classifier. The normalized ASV table (normalized to the lower number of sequence/sample) is then analyzed using Phyloseq (phyloseq object containing ASV table, taxonomic assignment, and environmental data) and Vegan package for diversity under R environment.

### Statistical analysis

Reporter cell lines were analyzed by analysis of variance (ANOVA) and a post-hoc test by Tukey’s honestly significant difference (HSD) test using JMP17 software. A *t*-test was performed to compare the treated conditions with the control on cell proliferation using R stat package. Results obtained from TEER, ELISA, and the *in vitro* fermentations were analyzed using Linear Model (lm function in R Stat package) and pairwise comparison using least significant difference (LSD) (LSD.test function in R Agricolae package). In addition, the *P* values were adjusted to account for multi-variable testing using false discovery rate (FDR) method for the phylum and genera tables obtained from 16S rRNA sequencing.

## Data Availability

Sequence files and metadata for all samples used in this study have been deposited at the DRYAD Dryad Digital Repository (doi: https://doi.org/10.5061/dryad.1rn8pk138).
